# Combining Biofilm-Controlling Compounds and Antibiotics as a Promising New Way to Control Biofilm Infections

**DOI:** 10.3390/ph3051374

**Published:** 2010-05-11

**Authors:** Andréia Bergamo Estrela, Wolf-Rainer Abraham

**Affiliations:** Helmholtz Center for Infection Research, Chemical Microbiology, Inhoffenstrasse 7, 38124 Braunschweig , Germany

**Keywords:** biofilm, microbial community, quorum-sensing, quorum-quenching, antimicrobial therapy

## Abstract

Many bacteria grow on surfaces forming biofilms. In this structure, they are well protected and often high dosages of antibiotics cannot clear infectious biofilms. The formation and stabilization of biofilms are mediated by diffusible autoinducers (e.g. *N*-acyl homoserine lactones, small peptides, furanosyl borate diester). Metabolites interfering with this process have been identified in plants, animals and microbes, and synthetic analogues are known. Additionally, this seems to be not the only way to control biofilms. Enzymes capable of cleaving essential components of the biofilm matrix, e.g. polysaccharides or extracellular DNA, and thus weakening the biofilm architecture have been identified. Bacteria also have mechanisms to dissolve their biofilms and return to planktonic lifestyle. Only a few compounds responsible for the signalling of these processes are known, but they may open a completely novel line of biofilm control. All these approaches lead to the destruction of the biofilm but not the killing of the pathogens. Therefore, a combination of biofilm-destroying compounds and antibiotics to handle biofilm infections is proposed. In this article, different approaches to combine biofilm-controlling compounds and antibiotics to fight biofilm infections are discussed, as well as the balance between biofilm formation and virulence.

## 1. Biofilm Infections Are Difficult to Treat Due to Complex Protection Mechanisms

Biofilms are microbial aggregations adhering to biological or non-biological surfaces. They can be found on any implant but also on the mucosa, e.g. in the cystic fibrosis lung [1], the middle ear [2] or gastric mucosa [3]. Biofilm formation is a key factor for microbial survival in hostile environments, representing a protected mode of growth that allows cells to survive and disperse [4]. Biofilm infections are a huge problem in the clinic and cause many deaths and high health costs, e.g. 12% to 25% of patient mortality are attributable to catheter-related bloodstream infections [5].

Biofilm infections are difficult to eradicate because the genetic program (gene expression) of bacteria in this structure is fundamentally changed [6] resulting in a much better protection against macrophages and antibiotics, compared to free living cells [7]. The eradication of *Escherichia coli* biofilms required 220 times higher antibiotic concentrations than for the same strain in serum [8]. The reasons for the higher antibiotic resistance of bacteria in biofilms are many fold and still not very well understood. In biofilms, up to 20% of all bacterial genes are expressed differently [9]. Bacteria grow slower and the reduced metabolic activity renders them less prone against most antibiotics. They also produce compounds actively detoxifying antibiotics, e.g. *Pseudomonas aeruginosa* produces only in biofilms a periplasmic cyclic glucan which complexes the antibiotics tobramycin and gentamicin, inactivating them [10]. This shows that the extracellular matrix of the biofilm is not only a passive diffusion barrier for antibiotics but is also actively shaped by species within the microbial biofilm communities. Interestingly, it has recently been reported that swarming also causes increased antibiotic resistances and that quorum-sensing is not involved in this effect [11].

## 2. Microbes Communicate by Autoinducers to Form Biofilms

Before forming biofilms, bacteria have to synchronize their gene expression in a process called quorum-sensing (QS) [12]. They do this by secreting small extracellular signal molecules acting as autoinducers to start genetic programs [13]. When a certain threshold of autoinducer concentration is reached, cells attach to the surface forming a biofilm and starting the production of virulence factors. Quorum-sensing is necessary both to start biofilm formation and, to a lesser extent, to maintain the biofilm. This process, however, is not absolute. It depends on the environmental conditions experienced by the cells and varies between the different species [14,15]. Iron is essential for most bacteria and its availability is often limited during the infection process. It has been shown that iron modulates the stress response in biofilms and overrides the expression of superoxide dismutases in *P. aeruginosa*, which is otherwise induced by QS [16]. Transcriptome results indicate that the QS system in *P. aeruginosa*, and probably in other bacteria as well, is far more complex than previously thought and many QS-regulated genes encoding virulence products were expressed in all conditions investigated [17]. In most pathogens studied so far, blocking QS does not abolish biofilm formation, but does make the biofilm more susceptible to antimicrobials and immune reactions.

**Figure 1:**
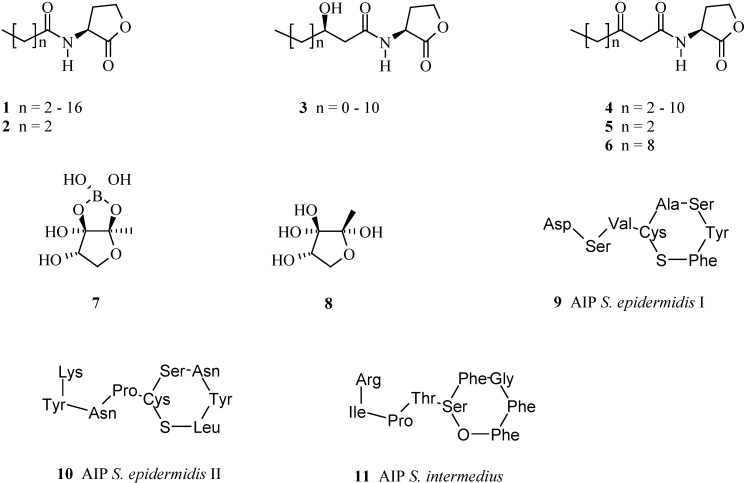
Small compounds, called autoinducers, used for quorum sensing and inducing biofilm formation.

Currently, more than 50 different autoinducers are known and the best studied ones, called autoinducer-1 (AI-1), are *N*-acyl-L-homoserine lactones (AHL) **1–6**, which are only known from Gram-negative bacteria. The acyl side chain of AI-1 has between 4 and 16 carbons and is usually saturated, although mono-unsaturated side chains are also known. AI-1 compounds **3–6** have also a hydroxyl or oxo-function at C-3 [18] (Figure 1). Another group of autoinducers, autoinducer-2 (AI-2), is known both from Gram-positive and -negative bacteria. The first AI-2 was found in *Vibrio harveyi* and identified as the cyclic boronic ester **7** [19]. Later, the epimer **8** lacking the boronic ester was detected in *Salmonella typhimurium* [20]. AI-2 is produced and detected by many bacteria and regarded as autoinducer for interspecies communication between bacteria [21]. A contradictory finding is that the only genes known to be regulated by the AI-2 system in bacteria other than *Vibrio* species encode the ABC transporter, LsrR, which is responsible for the uptake of the AI-2 signal in *S. typhimurium* and *Escherichia coli*. In the cell, AI-2 interacts with LsrR, which then represses the *lsr* operon [22,23]. This leaves the exact action and function of AI-2 in these organisms open, and a non-quorum sensing role for *luxS* in most bacteria has been suggested [24]. Such a non-quorum-sensing role of AI-2 has been demonstrated for *Staphylococcus aureus*. When grown together with the wild type in mixed culture, mutants of various *S. aureus* strains that are unable to produce AI-2 showed reduced ability to compete for growth under sulphur limitation. Inactivation of AI-2 production did not affect biofilm formation, nor virulence-associated traits, such as production of hemolysins and extracellular proteases. Interestingly, AI-2 production does not appear to contribute to the overall fitness of *S. aureus* during intracellular growth in epithelial cells [25].

Instead of acyl homoserine lactones (AHL), Gram-positive bacteria use small peptides as autoinducers (AIP). All peptide signalling molecules are formed by posttranslational modification of precursor peptides which are then actively secreted. Sensory information is relayed into the cell by phosphorylation cascades altering gene expression [26]. The cyclic octa- and nonapeptide AIPs from several *Staphylococcus* species are thiolactone peptides. Only the central cysteine and its distance to the C terminus are conserved but the primary sequence of the pheromones vary even in subgroups within one species [27,28]. For example, the AIPs **9** and **10** have been identified from *S. epidermidis* isolates [29]. The only exception is the AIP **11** of *S. intermedius* where the cysteine is replaced by a serine forming not the usual thiolactone, but a lactone. The AIPs produced by some strains of the same species inhibit other strains even of the same species, probably to exclude them from infection or colonization sites, or both [30].

Most bacteria live in multi-species microbial communities, therefore, the quorum-sensing signals are not only received and processed by cells of the same species, but also by foreign species [31]. This has been shown for *E. coli,* where both gene expression and phenotype alter in response to AHLs of foreign bacteria [32]. *Burkholderia cepacia* reacts to AHLs produced by *Pseudomonas aeruginosa*, but *P. aeruginosa* does not respond to *B. cepacia* AHLs [33]. The consequence is that *B. cepacia* always occurs together with *P. aeruginosa* in the cystic fibrosis lungs while *P. aeruginosa* is also found without a *B. cepacia* co-infection [34]. A much more complex autoinducer signal interaction concerning autoinducer AI-2 is found between the oropharyngeal flora and *P. aeruginosa* in cystic fibrosis. In this case, AI-2 is produced by the oropharyngeal flora and *P. aeruginosa* responds to it, although it does not produce AI-2 itself [35]. Additionally, antibiotic activity has been demonstrated for 3-oxo-dodecanoyl-AHL 6 and is directed against some Gram-positive bacteria, but was not observed for Gram-negative species [36].

## 3. Quorum-Quenching Is a Novel Target of Biofilm Control but Not the Only One

### 3.1. Natural products as leads for quorum-quenching drugs

**Figure 2:**
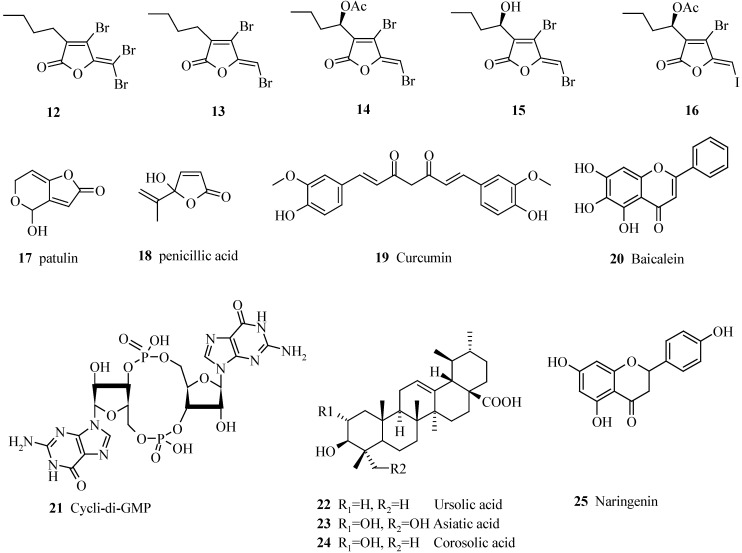
Natural compounds isolated from various organisms shown to interfere with biofilm formation or biofilm maintenance.

As all higher organisms are exposed to pathogens organized in biofilms, they have developed several strategies to control these biofilms. The red macroalga *Delisea pulchra* is devoid of biofilms and it has been speculated that this plant actively prevents biofilm formation. Several brominated furanones, **12–16**, have been isolated [37] and shown to interfere with the action of AHL in bacteria (Figure 2). These metabolites act in *Escherichia coli* by displacing *N*-3-(oxohexanoyl)-L-homoserine lactone 5 from its receptor LuxR [38]. To facilitate the access to these compounds, synthetic analogues, e.g. **29**, were synthesized, but most of them had less quorum-quenching ability than the natural metabolites [39,40]. It was shown that synthetic derivatives of the furanones target the QS regulon in *Pseudomonas aeruginosa* and inhibit virulence factor production as well [41]. The activity of **29** is not restricted to the interference with the AHL autoinducers, as it also inhibited *E. coli* biofilm formation via AI-2 [42]. By destroying the biofilm, the furanones reduced the virulence of *P. aeruginosa* in a mouse pulmonary infection model. The compounds promoted the clearance of *P. aeruginosa* by the mouse immune response and reduced the severity of lung pathology, resulting in prolonged survival of the infected mice [43,44]. One drawback, however, is that the halogenated furanone derivatives are probably too toxic for humans. An alternative to avoid side-effects related to toxicity is to search for biofilm-controlling compounds among herbal products, such as garlic (*Allium sativum*). In a mouse model of urinary tract infection with *P. aeruginosa* biofilms, oral administration of garlic extract resulted in reduced bacterial counts in the kidneys and attenuated inflammation of the renal tissue. *In vitro*, the extract was able to reduce biofilm formation and production of virulence factors like alginate, haemolysin and phospholipase C by *P. aeruginosa* [45]. In a clinical trial, garlic capsules were well tolerated. However, no significant effect of garlic compared to placebo was observed in this pilot study, even though suggestions of improvement with garlic were made, which should be investigated in a larger trial [46].

A number of other secondary metabolites have been shown to interfere with QS, but investigations are still in the beginning of drug development. Curcumin (**19**), a plant metabolite, causes at 1 µg L^-1^ a 25% reduction of 3-oxo-dodecanoyl-AHL **6** and a 2% reduction of butanoyl-AHL **2**, resulting in reduced *P. aeruginosa* pathogenicity [47] (Figure 2). Recently, the antibiofilm properties of citrus flavonoids were investigated. Among others, the flavanone naringenin (**25**) presented a prominent antagonistic activity against *E. coli* and *V. harveyi* biofilm formation, without affecting planktonic growth. Also, naringenin was shown to suppress the expression of type III secretion systems genes in *V. harveyi*, prompting this compound as a useful lead to antipathogenic drugs development [48].

### 3.2. Synthetic analogues as quorum-quenchers

**Figure 3:**
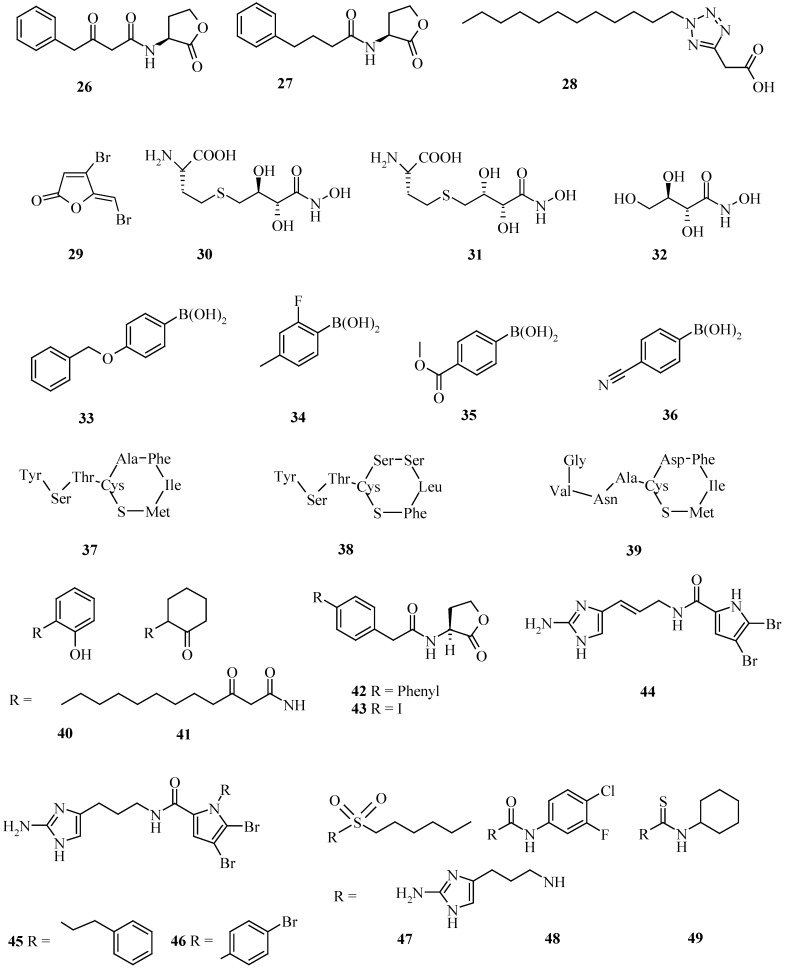
Synthetic compounds derived from autoinducers, or natural biofilm-controlling compounds as a lead or found in combinatorial chemistry libraries.

Natural inhibitors of QS, the structure of AHL itself, or precursors of AHL, were used as leads to synthesize quorum-quenching molecules. The phenyl-substituted AHL-analogues **26** and **27** (Figure 3) were antagonists of AHL (IC_50_ 2 μM) [49] and by ultra-high throughput screening, the tetrazole **28** with a C_12_-side chain was discovered as one of the most effective inhibitor reported so far (IC_50_ 30 nM) [50]. Several studies have revealed that small changes in a compound can turn it from an antagonist of AHL to an agonist. 

Among other aniline derivatives, compound **40** was found to be an antagonist of **6**. However, the derivative where the aromatic ring of **40** is saturated became a strong agonist [51]. The corresponding ketone **41** inhibit the AHL-synthesizing genes *lasl* and *rhlI*,causing reduction in pyocyanin, elastase and biofilm formation [52]. A number of benzyl derivatives of AHLs have been identified which could act as quorum-quenchers. It has been demonstrated that several of these biphenyl derivative, e.g. **42** and the 4-iodobenzyl compound **43**, could inhibit the third AHL receptor, QscR, in *P. aeruginosa* [53]. The same compounds also inhibited QS in *Vibrio fischeri* [54] and the bromo-derivative of **43** has been reported to strongly inhibit *P. aeruginosa* biofilm formation [55]. 

Most of these studies focused on one pathogen and reports of testing several pathogens with the same set of compounds are still rare. The pathogens *Agrobacterium tumefaciens*, *P. aeruginosa* and the model organism *V. fischeri* were tested with a library of about 90 compounds. Interestingly, the AHLs octanoyl- **1** (n = 6) and 3-ketodecanoylhomoserine lactone **4** (n = 6), as well as **43**, were quorum-quenchers for all three strains tested. In the same screen, compounds were identified which could specifically block QS in only one of the strains [56]. In 1971 the alkaloid oroidin (**44**) was reported from the sponge *Agelas oroides* [57] and later it was shown to modulate biofilm formation of a number of bacteria [58]. Oroidin was taken as a lead to synthesize a number of derivatives of which the sulphonamide **47**, the urea **48** and the thiourea **49** analogues were able to selectively inhibit *P. aerugionosa* biofilm formation, while being nontoxic to *C. elegans* [59]. Modification of oroidin led also to the derivatives **45** and **46**, all active against the pathogens *Acinetobacter baumannii*, *Pseudomonas aeruginosa* PA14 and *Bordetella bronchispetica* [60] (Figure 3).

The biosynthesis of AI-2 is the target for some AI-2-inhibitors. The enzyme LuxS catalyzes the cleavage of the thioether linkage in *S*-ribosylhomocysteine to produce homocysteine and 4,5-dihydroxy-2,3-pentanedione, the precursor of AI-27. Two hydroxamic acids turned out to be potent inhibitors of the enzyme, with K_I_ values below 1 μM. Their activity demonstrated nicely that not all LuxS of different species are similar. While the *erythro*-compound **30** is twofold less active than the *threo*-isomer **31** in *Bacillus subtilis* (0.72 *vs.* 0.37 μM), its activity is higher in *E. coli* (3.2 *vs.* 12.7 μM) and *V. harveyi* (9.7 *vs.* 12.8 μM). Compound **32**, where the homocysteinyl moiety is replaced by a hydroxyl group, is at least 50-fold less active [61]. The boronic acid analogues of AI-2, 4-(benzyloxy)- (**33**), 2-fluoro-4-methyl- (**34**), 4-(methoxycarbonyl)- (**35**) and 4-cyano-phenylboronic acid (**36**), had comparable activity, as shown by the inhibition of the luminescence of *V. harveyi* by quenching the AI-2 activity [62], (Figure 3).

### 3.3. Antagonists of AIPs

The thiolactone function of the AIPs is essential for activity in *S. aureus* and can only be partially replaced by the corresponding lactone or lactam [63]. The hydrophobic patch on the AIPs is required for binding to the receptor and is an absolute requirement for interaction [64]. Replacement of the endocyclic aspartic acid with an alanine residue converted the AIP from a group I strain activator to a potent inhibitor **37** (IC_50_ 33 nM) [65]. The chimera **38** and **39**, constructed by mixing elements of several AIPs, universally inhibited AgrC activation [66]. Although there are many optimistic reports on the application of quorum-quenching AIPs, none of them made it to clinical trials. The main reason may be that the role of AIP in biofilm formation and expression of virulence factors is complex. The agr (accessory gene regulator) quorum-sensing system of *S. aureus* modulates the expression of virulence factors in response to AIPs. To form a biofilm, repression of agr is necessary and its reactivation triggers biofilm detachment. It is tempting to block activation of virulence gene expression by blocking QS, however, as in the case of staphylococci, the agr system can regulate gene expression both positively and negatively [67]. Animal and clinical studies have revealed other problems; e.g. agr mutants are frequently found in clinical isolates and some strains alternate between an agr positive and agr negative phenotype, making the development of resistance highly likely. Also, due to the specificity of AIPs, suppressing one type of pathogen may benefit more invasive strains. More important however is that agr inactivation has been associated with increased antibiotic resistance, suggesting that sometimes activation rather than inhibition of the agr response may have therapeutic benefits.

## 4. There Are More Ways to Control Biofilms Than Interference with the Quorum-Sensing System

Quorum-sensing is currently regarded as a key mechanism in biofilm development and the wealth of autoinducers offer an attractive new target for non-antibiotic control of biofilm infections [68]. The sophisticated action of autoinducers in different bacteria species, the existence of several QS-control mechanisms in one cell, and the complex interaction between autoinducers of different species, demonstrated that the goal of controlling biofilm infections is not easily achievable. Obviously, searching for biofilm control beyond the interference with the known QS-autoinducers seems to be an attractive goal.

### 4.1. Fatty acids acting as diffusible signal factor

**Figure 4:**
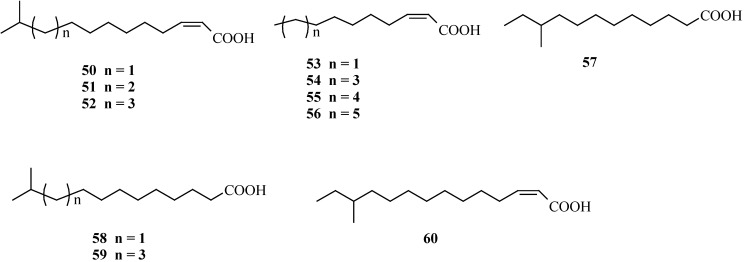
Fatty acids isolated from various bacteria and acting as diffusible signal factor in communication with other species.

Several fatty acids have been identified acting as the signalling molecules between bacteria species regulating virulence factors of the recipient and were termed diffusible signal factors (DSF) [69]. From *Stenotrophomonas maltophila*, an opportunistic pathogen difficult to treat by antibiotics [70], eight fatty acids (**50–52**, **55–58**, **60**) have been identified which facilitate movement of its cells [71] and mediate the communication within the species and *Pseudomonas aeruginosa*. The fatty acids produced by *S. maltophila* promote increased stress tolerance in *P. aeruginosa* and increased resistance against cationic antimicrobial peptides [72]. *P. aeruginosa* has probably interactions via DSF to other bacteria as well, because 12-methyl-tetradecanoic acid **59**, not found in *S. maltophila*, inhibited swarming motility completely and reduced biofilm formation by 31% at 10 µg mL^-1 ^[73]. *cis*-2-Dodecenoic acid **54** mediate the communication between *Burkholderia cenocepacia* and *Candida albicans* [74] (Figure 4). These findings show that DSFs similar to AHLs seem to have a rather dedicated role in bacteria-bacteria and even bacteria-fungus interaction, and any medicinal applications for biofilm control will probably focus on the action on a specific pathogen. From the supernatant of spent cultures of *P. aeruginosa*, a small molecule acting as dispersion autoinducer was isolated. The compound has been reported to be effective in dispersing biofilms containing not only *P. aeruginosa*, but also *Streptococcus mutans*, *Escherichia coli*, and *Staphylococcus aureus*, regardless of whether the bacteria exist in a pure or mixed-culture biofilm [75]. The structure of this compound has recently been reported and it turned out to be **53**, another fatty acid of the DSF type [76]. When added to *P. aeruginosa* PAO1 biofilms at a native concentration of 2.5 nM, *cis*-2-decenoic acid (**53**) induced the dispersion of biofilms. This molecule was also shown to induce dispersion of biofilms formed by *E. coli*, *Klebsiella pneumoniae*, *Proteus mirabilis*, *Streptococcus pyogenes*, *Bacillus subtilis*, *S. aureus*, and the yeast *C. albicans*. This result is surprising because structurally related DSFs are hitherto reported to act rather specifically on certain microorganisms. 

### 4.2. Nitric oxide (NO) can disperse biofilms

Nitric oxide has been identified to disperse *P. aeruginosa* biofilms [77] but is also active in Gram-positive pathogens. Nitrite-induced stress reduced polysaccharide intercellular adhesin synthesis in *Staphylococcus aureus* and *S. epidermidis* and led, as a consequence, to reduced biofilm formation. The addition of nitric oxide (NO) scavengers prevented the reduction of biofilm formation and identified NO as the active molecule in this process in *S. aureus* [78]. Silica nano-particles releasing NO have been shown to be an alternative to a NO-releasing coating. Viability experiments revealed that 99% of cells of *P. aeruginosa*, *E. coli*, *S. aureus*, *S. epidermidis*, and *C. albicans* biofilms were killed and/or dispersed via NO release. Cytotoxicity tests, however, demonstrated as well that the highest dose of NO-releasing silica nanoparticles inhibited fibroblast proliferation to some extent [79]. NO-releasing coatings on implants reduced implant-associated *S. aureus* infections in a rat model [80]. Nitric oxide interacts with the bacterial second messenger nucleotide cyclic di-GMP **21** [81]. A review on the role of cyclic di-GMP in pathogenesis appeared recently [82]. However, the role of cyclic di-GMP is still debated and it has been demonstrated to have an ambivalent role, since it promotes biofilm formation at high intracellular concentrations, whereas low levels lead to motility and the synthesis of virulence factors in pathogens. Studies of *P. aeruginosa*, however, did not corroborate this general view [83]. One has to take into account here that most of the results comes from few well-established model pathogens, including *Pseudomonas, Vibrio*, and *Salmonella*, while the mechanisms in the vast majority of bacteria involved in clinical infections are still largely unknown [84]. 

### 4.3. Hydrolysis of extracellular DNA (eDNA)

In biofilms, microbial cells are embedded in a complex mixture of exopolysaccharides, proteins, and DNA. Some bacteria, including *P. aeruginosa*, excrete substantial quantities of extracellular DNA (eDNA). This eDNA seems to have an important function for the stability of the biofilm, because addition of DNase I leads to the dissolution of the biofilms. Application of DNase I is used to treat *P. aeruginosa* infections of the cystic fibrosis (CF) lung as a therapy to reduce the viscosity of purulent sputum. The discovery of eDNA suggest that this treatment is probably also beneficial to weaken biofilms in chronic *P. aeruginosa* infections [85]. Formation of eDNA is not limited to *P. aruginosa*. Also *Staphylococcus aureus* and *S. epidermidis* were found to excrete DNA into their biofilm matrix and it has been shown that eDNA of *S. epidermidis* is similar to its chromosomal DNA [86]. The role of eDNA, however, is not the same for all bacteria, and its role can be quite different in species within the same genus. This has been demonstrated by the addition of DNase I to *S. aureus* and *S. epidermidis*. DNase I inhibited biofilm formation in both species, but only *S. aureus*, and not *S. epidermidis* biofilms were dissolved and sensitized to killing by the cationic detergent cetylpyridinium chloride [87]. 

## 5. Dispersion of the Biofilm Can Increase the Virulence

There are several reports stating that the virulence of some pathogens is reduced when they are growing in biofilms. At the ASM Biofilms 2009 conference in Cancun, Mexico, David Davies presented data showing that the application of the biofilm dispersing factor 2-*cis*-decenoic acid (**53**) to *Pseudomonas fluorescens* infected leaves led to the dispersion of the biofilm on the one hand, but also to an enhanced virulence of the pathogen [88]. Further evidence comes from studies of the type III secretion systems (T3SS) in many bacteria, translocating pathogenicity factors into eukaryotic host cells. For *P. aeruginosa*, a balance was reported between active repression of the T3SS, leading to the establishment of *P. aeruginosa* in biofilms, and expression of T3SS in these biofilms, leading to the formation of virulence factors [89]. In contrast to these results, *P. aeruginosa* small colony variants, isolated from chronically infected cystic fibrosis patients, have both an increased capacity to form biofilms and increased T3SS gene expression and cytotoxicity [90]. The report that planktonic cells do not express T3SS, but cells in biofilms do, underlines the fact that the relations between biofilm mode of growth and T3SS expression is still not completely understood [91]. It seems that a homeostasis exists which might contribute to the persistence of chronic infections. As a consequence, biofilm repression might lead to an activation of T3SS and increased export of virulence factors. 

In *S. aureus*, sarZ deletion mutant clones presented increased expression of SarA protein and a strong down-regulation in agr RNAIII transcription, both important components of the virulence gene regulation in staphylococci. Accordingly, the expression of virulence factors (alpha-hemolysin and serine protease sspA) in these mutants is markedly reduced, while the biofilm formation ability is actually enhanced. The results suggest the promotion of virulence by SarZ includes repression of sessile bacteria establishment in biofilms, hence maintaining active *S. aureus* infections [92].

Another interesting mechanism by which bacteria can prevent eradication of infection is called indirect pathogenicity. In this polymicrobial interaction, an antibiotic-sensitive pathogen can be commensally protected by resistant bacteria of low intrinsic virulence. Studies in a model using *Escherichia coli* strains differentially susceptible to antibiotics showed that the biofilm environment limited the beneficial interaction between the strains in ampicillin-containing medium, the decline of susceptible population being two orders of magnitude lower in planktonic cultures than in biofilms [93].

These reports demonstrate that merely dissolving biofilms may not be sufficient to control biofilm infections. Nevertheless, quorum-sensing systems are important targets to address the sensitivity of bacteria to antibiotic compounds and the host immune system itself. This was demonstrated, for example, using *P. aeruginosa* deletion mutants for QS-receptor (ΔlasR rhlR). Mutant biofilms were significantly more sensitive towards reactive oxygen and polymorphonuclear leukocytes activity, being more rapidly cleared in an *in vivo* model of lung infection. Also, QS-deficient biofilms were more susceptible to killing by tobramycin [94]. Although it might be enough to dissolve the biofilm, attenuating the antibiotic resistance of the pathogens to the levels of single cells and leaving the rest to the immune reaction of the host, it is more advisable to support this process by a suitable antimicrobial therapy. 

## 6. Combining Quorum-Quenching Compounds with Antibiotics Can Treat Biofilm Infections

Dissolving pathogenic biofilms exposes the bacteria to the immune system of the host. It may be possible that the immune reaction is sufficient to clear the pathogen, but it is more likely that some bacteria escape the immune response and establish again biofilms. To address this problem, several attempts have been made to kill the bacteria released from the biofilm by treatment with standard antibiotics. In a pulmonary model of chronic lung infection ,the quorum-quenching furanone **29** was administered to mice infected with *Pseudomonas aeruginosa* two days previously. Attenuation of virulence factor expression and much better clearance of the bacteria by the immune system were observed [95]. From the fungus *Penicillium*, patulin (**17**) and penicillic acid (**18**) were identified as quorum-quenching compounds. Furthermore, both compounds activate polymorphonuclear neutrophils, breaking the blockage of the oxidative burst. When patulin (**17**) and penicillic acid (**18**) were tested in the mouse model, results similar to the furanone **29** were observed [96]. All three compounds increased the sensibility of *P. aeruginosa* to tobramycin, opening another window to control this bacterium in the lung of cystic fibrosis patients. 

The triterpene ursolic acid (**22**) was found to inhibit biofilm formation in *Escherichia coli, P. aeruginosa* and *Vibrio harveyi*, (10 µg mL^-1^) when added upon inoculation or to a 24-h biofilm, but it does not interfere with quorum sensing, as shown with *V. harveyi* AI-1 and AI-2 reporter systems [97]. The closely related asiatic acid (**23**) and corosolic acid (**24**) are even more effective and enhance the susceptibility of *P. aeruginosa* to antibiotics [98] (Figure 2). This activity is limited to biofilms younger than 24 h; however, biofilm infections are usually caused by much older biofilms, limiting any application of these compounds in medicine.

In *Agrobacterium tumefaciens*, TraR is the receptor for AHLs. A computer-directed search using the 3D-structure of TraR for natural compounds fitting into the receptor pocket identified, among other compounds, baicalein (**20**) as a possible candidate. Baicalein could indeed dissolve *P. aeruginosa* biofilms and showed synergistic effects with ampicillin, causing eradication of *P. aeruginosa* biofilms at 2 μg mL^-1^, a concentration not effective to kill the bacteria without baicalein [99]. No *in vivo* experiments have been reported and it is not known whether baicalein in combination with ampicillin can also eradicate old *P. aeruginosa* biofilms, e.g. in the lung of cystic fibrosis animals. 

As described above, low concentrations of NO cause *P. aeruginosa* biofilms to disperse, converting the bacteria in the planktonic phase to their normal susceptibility to antibiotics. This led to the suggestion that a combined exposure to both NO and antimicrobial agents may be a way to control pre-established *P. aeruginosa* biofilm infections [100]. A similar approach has been suggested by Melander *et al.*, who combined the anti-biofilm activity of a synthetic compound with photodynamic inactivation involving a suitable dye to kill *Acinetobacter baumannii* biofilms [101]. The same group reported on a dramatically increased sensitivity against conventional antibiotics when biofilms of multi-resistant *Staphylococcus aureus* (MRSA) or *A. baumannii* were treated with biofilm-controlling 2-aminoimidazoles in combination with antibiotics [102].

The enzymatic activity of the β-*N*-acetyl-glucosaminidase dispersin B is able to dissolve mature biofilms of *S. epidermidis*. As a promising tool for preventing biofilm colonization in medical devices, dispersin B was shown to directly decrease biofilm formation in polyurethane surfaces, and most importantly, to enhance the activity of the antibiotic cefamandole nafate against adherent cells in these surfaces [103].

## 7. Conclusions

The prevalence of bacterial pathogens living in biofilms, where they are much more resistant to antibiotics and clearance by the immune system, has promoted the search for new strategies to control biofilm infections. The discovery that bacteria have to coordinate their genetic programs to form a biofilm, and that they use small molecule autoinducers to do so, led to the search for quorum-quenching drugs. Impressive progress has been made in this area, revealing the huge diversity of autoinducers and their mechanisms of action, and convincing *in vitro* results have been published. However, none of the compounds made it yet to the clinical phase II stage. One problem is that many compounds can prevent the formation of biofilms but are not effective in dissolving existing ones and are, therefore, of little use for the treatment of already existing biofilm infections. Furthermore, for some compounds, even merely the concentration can switch between agonizing and antagonizing quorum-sensing [104]. In addition, redundancies among QS systems of several pathogens can render inhibitors of a single system ineffective, as has been shown e.g. for the AHLs of *Pseudomonas aeruginosa* or the AIPs of *Staphylococcus aureus*. Most of these studies focus on few, well studied pathogens [105] but most bacteria occur in complex microbial communities. An advantage for pathogens in microbial communities is that the communication advances to a complex interspecies cross-talk, favouring cheating, where single cells do not bother to take the metabolic burden to produce the autoinducer, but take it from the neighbour. To deal with such new challenge for drug development, other components for biofilm maintenance than quorum-sensing have been explored, opening novel strategies for control. Furthermore, several studies have revealed a balance for pathogenicity and the biofilm mode of growth [106]. 

The two problems: high specificity of autoinducers combined with a complex network of—often redundant—regulation circuits, and an increased virulence of pathogens released from biofilms, are real challenges in the development of quorum-quenching compounds for medical applications. As a consequence, many researchers have combined quorum-quenching with antibiotic treatments and demonstrated in animal studies that it worked well. This approach is based on the observation that most quorum-quenching compounds enhance the sensitivity of pathogens to antibiotics, even if the quorum-quenchers could not achieve complete dissolution of the biofilms. In animal models, this strategy of combination has already been successfully validated and could overcome many shortfalls of the application of quorum-quenching or other biofilm-controlling compounds alone. Such a combination is attractive and holds high expectations, but one should also bear in mind that we are dealing here with drugs influencing each other and complicating pharmacokinetics. Future biofilm infection treatment will probably use sophisticated drugs tailored to the causing pathogen and comprising both biofilm-controlling compounds and antibiotics.
